# Participation-based clinical clerkships contribute to increased medical student confidence in community emergency care: a cohort study

**DOI:** 10.1186/s12909-025-07317-1

**Published:** 2025-05-20

**Authors:** Hiroshi Mihara, Atsushi Jinno, Kenta Sato, Kazuhito Nomura, Takao Wakabayashi, Yoshihisa Tsuji

**Affiliations:** 1 Department of Educational Development, Center for Medical Education, Sapporo Medical University, S1 W17, Chuo-ku, Sapporo, 060-8556 Hokkaido Japan; 2 Department of General Medicine, Faculty of Medicine, Sapporo Medical University, Sapporo, Hokkaido Japan; 3 Division of Internal Medicine, Department of Internal Medicine, Chitose City Hospital, Chitose, Hokkaido Japan; 4 Department of General and Emergency Medicine, Japan Community Healthcare Organization Sapporo Hokushin Hospital, Sapporo, Hokkaido Japan

**Keywords:** C-CEP (Community-based assessment for clinical and emergency practice ) scale, Clinical clerkship, Confidence in community and emergency practice, Text mining

## Abstract

**Background:**

The Community-based assessment for Clinical and Emergency Practice (C-CEP) scale is a validated tool comprising 15 items across four components: “Attitude and Communication in Emergency Medicine,” “Basic Clinical Skills,” “Knowledge about Community Medicine,” and “Knowledge in Evidence-Based Medicine.” The C-CEP scale enables medical students’ self-assessment of their confidence in clinical and emergency care. This study aimed to evaluate whether a two-week comprehensive general practice clerkship would improve C-CEP scores and to identify the specific training elements contributing to increased confidence in primary care, particularly in emergency responses.

**Methods:**

After obtaining ethical approval and written informed consent, fifth-year medical students at Sapporo Medical University who participated in two-week off-campus clerkships at external healthcare facilities (hospitals) during the 2023 academic year were included. Pre- and post-clerkship C-CEP scores were collected. Changes in the baseline and average scores were analyzed across facilities accepting more than four trainees. Text mining was conducted on students’ reflective portfolios from the facilities with significant findings to identify frequent and co-occurring words related to confidence-building activities.

**Results:**

Out of 115 eligible participants, 97 students were included in the analysis (84.3% participation rate). The data from seven facilities accepting more than four trainees, representing 74 students in total, were analyzed. These facilities averaged 243 beds, were located 147 km from the medical school, and had an emergency admission rate of 84.6%. C-CEP scores showed significant improvement across all components post-clerkship (*p* < 0.05). Although pre-clerkship scores did not differ significantly across facilities, post-clerkship score increases varied. Multivariate analysis identified Facilities D and F as exhibiting significant differences in confidence improvement compared with the other facilities (R² = 0.20, *p* = 0.0379). Text mining highlighted that Facility F emphasized active learning activities such as “interviews,” “examinations,” and “conferences,” whereas Facility D focused on passive activities such as “observation”, which were associated with limited impact on trainee confidence.

**Conclusion:**

The C-CEP effectively measured confidence improvements in primary care and emergency responses among fifth-year medical students following their clinical clerkships. The C-CEP scale’s ability to detect inter-facility differences highlights its potential to inform and refine clinical education programs, ensuring more targeted and effective training.

**Supplementary Information:**

The online version contains supplementary material available at 10.1186/s12909-025-07317-1.

## Background

The uneven distribution of physicians, particularly the shortage of doctors in rural areas and underserved regions, poses a significant challenge to healthcare systems worldwide. This inequitable distribution perpetuates health disparities and leads to reduced access to medical services and worsened health outcomes for residents of underserved areas [1]. To mitigate these shortages, many nations have introduced preferential admission criteria, rural training programs, and financial incentives; however, their success hinges on the social identities and motivations of applicants and is often constrained by funding, regulatory barriers, institutional resistance, and issues of long-term sustainability [2–4].

Educational interventions, such as clinical training in rural settings, have been shown to significantly improve students’ patient-centered care skills. By interacting with patients in primary care facilities, students acquire practical skills to address real-world health challenges [5–7]. Medical schools have integrated rural health topics into their curricula and established rural medicine specialty tracks to prepare students for the unique challenges of practicing in these environments. Additionally, the integration of interprofessional education (IPE) and rural health education has been proposed [8–11]. Evidence indicates that rural clinical training enhances medical students’ patient-centered care skills, understanding of community health, and interprofessional collaboration capabilities. Students who experience rural training tend to exhibit higher motivation to pursue careers in rural healthcare after graduation, making rural training a crucial tool for teaching students how to address community health challenges.

The willingness to engage in rural healthcare appears to be rooted in students’ confidence in their own clinical skills, as supported by previous studies showing that higher self-efficacy in rural medical practice is associated with stronger intentions to pursue rural careers [12, 13]. Regarding students’ confidence in their clinical competencies, while clinical training improves students’ confidence in their clinical competencies, a report indicated that the location of the placement (urban, suburban, or rural) does not significantly influence the degree of confidence [14]. This suggests that factors other than placement location may contribute to confidence building, highlighting the importance of identifying these factors. Thus, rural healthcare education and practice, which deepen students’ understanding of and confidence in rural healthcare, may promote career choices in rural medicine and consequently increase the number of healthcare professionals in rural areas [1, 6].

To further explore these challenges, there is a need for self-assessment tools that can capture aspects of learner development not addressed by traditional evaluations. Standard measures, such as written exams or clinical task logs, often fail to assess students’ subjective sense of preparedness and their confidence in applying knowledge to real-world settings. Since confidence is closely tied to motivation, clinical judgment, and career orientation, especially in underserved areas, tools like the C-CEP can fill this evaluative gap and provide actionable insights for improving clinical education. to evaluate the effectiveness of educational programs objectively and accurately. To address this need, we developed and validated a self-assessment tool, the Community-based assessment for Clinical and Emergency Practice (C-CEP), for medical students in the context of rural and emergency medicine [15] (Supplementary Table 1). This tool can be applied to measure changes in students’ confidence before and after primary care clinical training and to identify factors that enhance their confidence in rural healthcare. To avoid potential confusion between “community care” and “rural medicine,” it is important to clarify that in this study, “community care” refers to clinical experiences conducted at off-campus, non-tertiary facilities. These facilities are located outside the main university campus but do not necessarily meet standard definitions of “rural” based on population size or geographic remoteness. In this study, we use the term “primary care clinical training” to refer to educational experiences provided at non-tertiary, community-based facilities, many of which are located in rural or semi-rural settings. While “primary care” denotes a level of care focused on first-contact, comprehensive, and continuing services, it often overlaps with “rural healthcare” in practice due to the nature of training environments situated outside urban academic centers. Therefore, although the background of this study emphasizes rural healthcare, the scope of training described includes both the functional aspects of primary care and the geographical aspects of rural medical practice. The focus of this study is therefore on community-based experiential learning, rather than evaluating outcomes related to rural health workforce distribution. This study aimed to evaluate whether rural clinical training improves medical students’ confidence, addressing the educational gap of lacking tools to monitor subjective changes in learner confidence—particularly in the context of rural placements in rural healthcare, including emergency care, as assessed by their C-CEP scores, and to identify the attributes of clinical training and education that contribute to increased confidence in rural healthcare.

## Methods

### Study design and participants

This cohort study was conducted among fifth-year medical students participating in two-week clinical clerkships at external healthcare facilities as part of the General Medicine program during the 2023 academic year at Sapporo Medical University. Students were placed at 21 non-tertiary community facilities, varying in geography and demography (e.g., small towns and peri-urban settings), with each facility hosting approximately 2–6 students depending on capacity. Facilities were assigned based on availability and preferences. In the context of this study, “community facilities” are defined as off-campus, non-tertiary care facilities where students engage in clinical clerkships. These facilities vary in their geographic and demographic profiles, including small towns and peri-urban locations, and are not necessarily classified as “rural” by national standards.

### Educational Interventions and Clinical Training Structure

The two-week clerkship aimed to foster the following learning objectives:

- Actively participate in daily activities of physicians and multidisciplinary medical staff in non-university, community-based facilities.
- Reflect on clinical experiences and emotions through the creation of a daily portfolio.
- Develop comprehensive clinical capabilities essential for general practitioners working in community settings, including understanding the multifaceted social and health-related challenges faced by patients, families, and communities.

The competencies addressed included professionalism, medical knowledge, clinical practice, communication, EBM-based problem-solving, and interdisciplinary collaboration. Instructional methods were aligned with each facility’s local context and included bedside learning, outpatient care, home visits, and interprofessional case conferences. At the end of the clerkship, students submitted a portfolio and participated in a final group debriefing.

### Ethical considerations

Personal identifiers were anonymized from the C-CEP scales and portfolios prior to analysis. Participation was voluntary, and only data from students who consented were included. All students provided informed written consent to participate in the study.

### Educational interventions and data collection procedures

Formative assessments included Mini-CEX (used at some sites), 360-degree evaluations, and required portfolio submission. The portfolios were written in Japanese and followed a daily reflection format, assessing content depth, specificity, and personal insight. Translations were conducted by the authors and reviewed through academic proofreading. Notably, the term “見学” was automatically translated to “inspection” in word cloud analysis; this was manually corrected to “observation” to reflect clinical meaning.

Students completed the C-CEP scale before and after the program. During their clerkships, the students maintained portfolios documenting their daily experiences, reflections, and learning outcomes. Portfolios followed the Significant Event Analysis (SEA) model, emphasizing significant realizations, accomplishments, challenges, and emotional responses. Feedbacks were provided by supervising physicians, which were not included in the analysis.

### Quantitative analysis of C-CEP scores

We used the validated 15-item version of the C-CEP without modification [15] (Supplementary Table 1). This self-assessment scale employs a 1–5 Likert scale (1– strongly disagree; 5– strongly agree) and consists of four domains: “Attitudes and Communication in Emergency Care” (items 1–3), “Basic Clinical Skills” (items 4–7), “Knowledge Related to Community Healthcare” (items 9–13), and “Knowledge in Evidence-Based Medicine” (items 8, 14, 15). The scale has demonstrated strong psychometric properties, including high internal consistency (McDonald’s ω ≥ 0.70) and excellent model fit indices (CFI = 0.979, RMSEA = 0.045) in prior validation studies. In this study, changes in total and domain-specific C-CEP scores before and after the clerkship were analyzed using paired t-tests (*p* < 0.05). For facilities that accepted four or more students during the study period, contextual variables were included in multivariate linear regression models: number of facility beds, emergency acceptance status, distance from Sapporo Medical University, geographic location, and the population of the surrounding city. To reduce selection bias, all eligible facilities were analyzed regardless of their emergency care capacity. As a result, Facility C—despite not accepting emergency cases—was included in the dataset. Group comparisons were further informed by exploratory t-tests to identify optimal cut-off points for explanatory variables. Boxplots were used to visualize score distributions, highlighting not only central trends but also the presence of outliers and students who experienced decreased confidence post-clerkship. In the multivariate linear regression model, the variable “facility” was treated as a categorical factor using dummy coding. Facility H was set as the reference category; therefore, estimates for Facility H are not explicitly displayed in the model output, and all other facility coefficients represent differences relative to Facility H.

### Qualitative text mining of reflective portfolios

Portfolios were text-mined using AI-based software (https://www.userlocal.jp) to analyze frequent words (nouns and verbs) and word co-occurrence patterns [16, 17]. The text mining results from trainees at facilities with notable C-CEP outcomes (e.g., Facilities D and F) were compared to identify reflective language differences. Text mining focused on noun and verb extraction from Japanese portfolios, with separate analyses for students at Facilities D and F. Additionally, word clouds were generated from the processed texts to intuitively visualize frequently used terms. These word clouds did not rely solely on raw frequency counts, but instead used frequency scores adjusted to exclude high-frequency, low-informational general terms (e.g., “do,” “think,” “go”). This refinement allowed for a more meaningful representation of content-relevant expressions. Word clouds were generated based on frequency scores, excluding low-informational general terms. Corrections were made for key translation errors during the English rendering process. These visualizations offered insights into differences in reflective language and learning emphasis across institutions. Separate visualizations were created for Facilities D and F to highlight institutional differences in reflective language patterns. Text mining provided insights into the linguistic patterns associated with the portfolio reflections, facilitating a deeper understanding of the educational impact of the clerkships.

### Statistical tools

All statistical analyses, including t-tests, one-way ANOVA for univariate comparisons involving three or more groups, and multivariate regression modeling were conducted using JMP^®^ Pro 17.0.0, with *p* < 0.05 considered to be significant.

## Results

### Participant demographics

Out of 115 potential participants in the general practice clinical education program, 97 students were included in the analysis (84.3% participation rate). The average age of the participants was 23.5 years, and 70.1% were male (Table 1).

**Table 1:** Demographic characteristics of the student Doctors

*N*	Age, years (mean)	Male (%)	Participation rate (%)
97	23.5	70.1	84.3 (97/115)

### C-CEP scores before and after clerkship

**Fig. 1:**
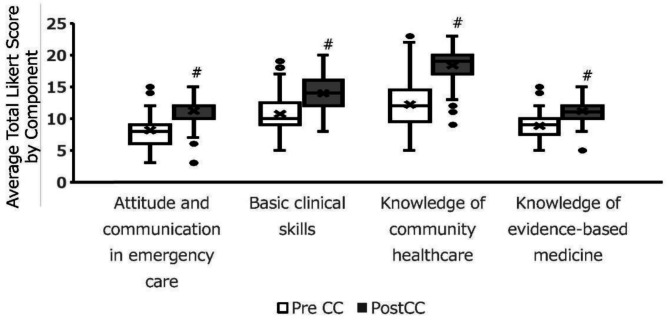
Comparison of the average total Likert scores for the four C-CEP components before and after the general medicine clinical clerkship (*n* = 97). Statistically significant changes (*p* < 0.05) are indicated by #. Boxplots visualize the distributions of total and domain-specific C-CEP scores, highlighting medians, interquartile ranges, and outliers

**Fig. 2:**
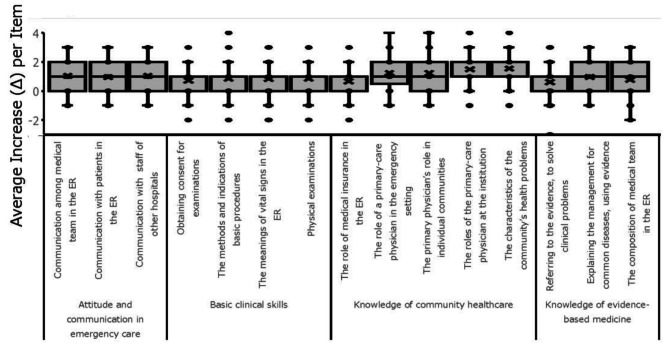
Boxplot comparing changes in scores (average increase per item; D) for all 15 C-CEP questions before and after the general medicine clinical clerkship across different facilities (*n* = 97). The plots show the median, interquartile range, and outliers for each institution. Notably, some students exhibited score decreases, indicating that the clerkship experience may have led to reduced confidence in specific domains. ER, emergency room

The average C-CEP scores across all four components showed significant increases after the two-week clerkship (*p* < 0.05, Fig. 1). The degree of improvement for individual items within the components varied (Fig. 2). Figures 1 and 2 present boxplots showing the distributions of pre- and post-clerkship C-CEP scores across domains. Items such as “The roles of the primary-care physician at the institution” and “The characteristics of the community’s health problems” demonstrated a notable average increase of > 1.5, whereas “The role of medical insurance in the emergency room” showed a smaller average increase of 0.5. Notably, the boxplot in Fig. 2 revealed that while the majority of students demonstrated an increase in C-CEP scores, a subset of students showed a decline in confidence after the clerkship. This variability suggests that clinical exposure may not uniformly enhance self-assessed competencies and may, in some cases, lead to a more critical self-appraisal.

### Facility and trainee information

Facilities that accepted four or more trainees (Facilities A-H) and the associated trainee demographics are summarized in Table 2. The facilities averaged 243 beds (range 0–498; SD 149), with an emergency acceptance rate of 84.6% (11/13), were located an average of 147 km from Sapporo Medical University (range 0–370 km; SD 124 km), and served an average population of 600,000 (range 10,000–1,958,000; SD 836,000). Facilities A and G accepted a higher number of trainees, whereas facilities B, E, and H had a higher proportion of female trainees. Facility D had a relatively low participation rate.

**Table 2:** Characteristics of the facilities (hospitals) hosting trainees for their clinical clerkships

	Facility				Students
	Number of beds	ER (present: 1, absent: 0)	Distance from medical school (Sapporo) (km)	Population of city/town (thousands)	*n*
A	< 200	1	> 200	< 30	19
B	< 200	1	< 100	< 30	11
C	< 200	0	> 200	< 300	4
D	> 200	1	100–200	< 30	9
E	> 200	1	100–200	< 300	6
F	> 200	1	0	> 300	8
G	> 200	1	< 100	< 30	12
H	< 200	1	> 200	< 30	5

### Facility-specific effects in clinical clerkship: a multivariate analysis of C-CEP score improvements

**Fig. 3:**
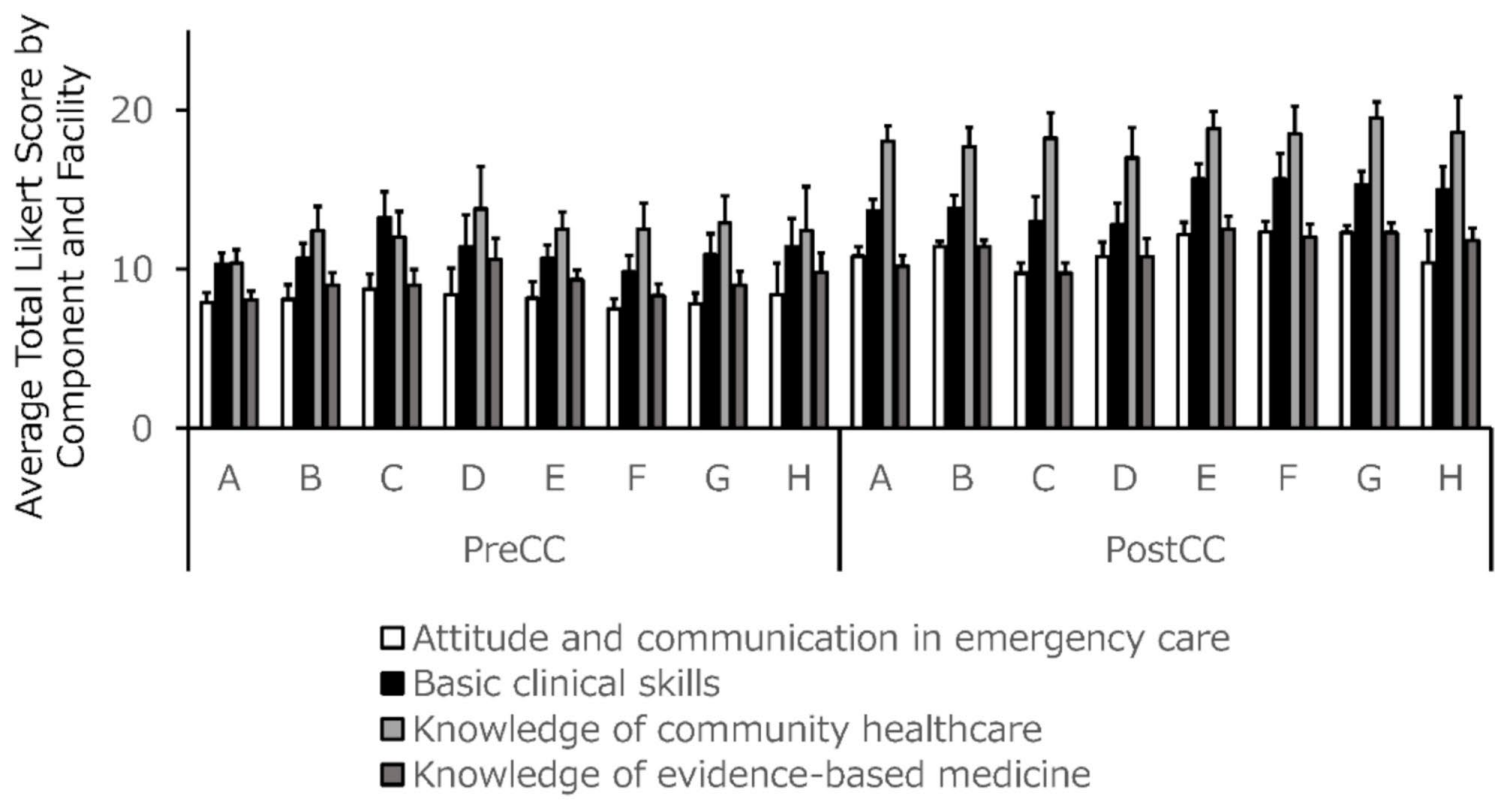
Average total Likert scores for the four C-CEP components before and after the clinical clerkship, by facility (facilities A to H)

**Fig. 4:**
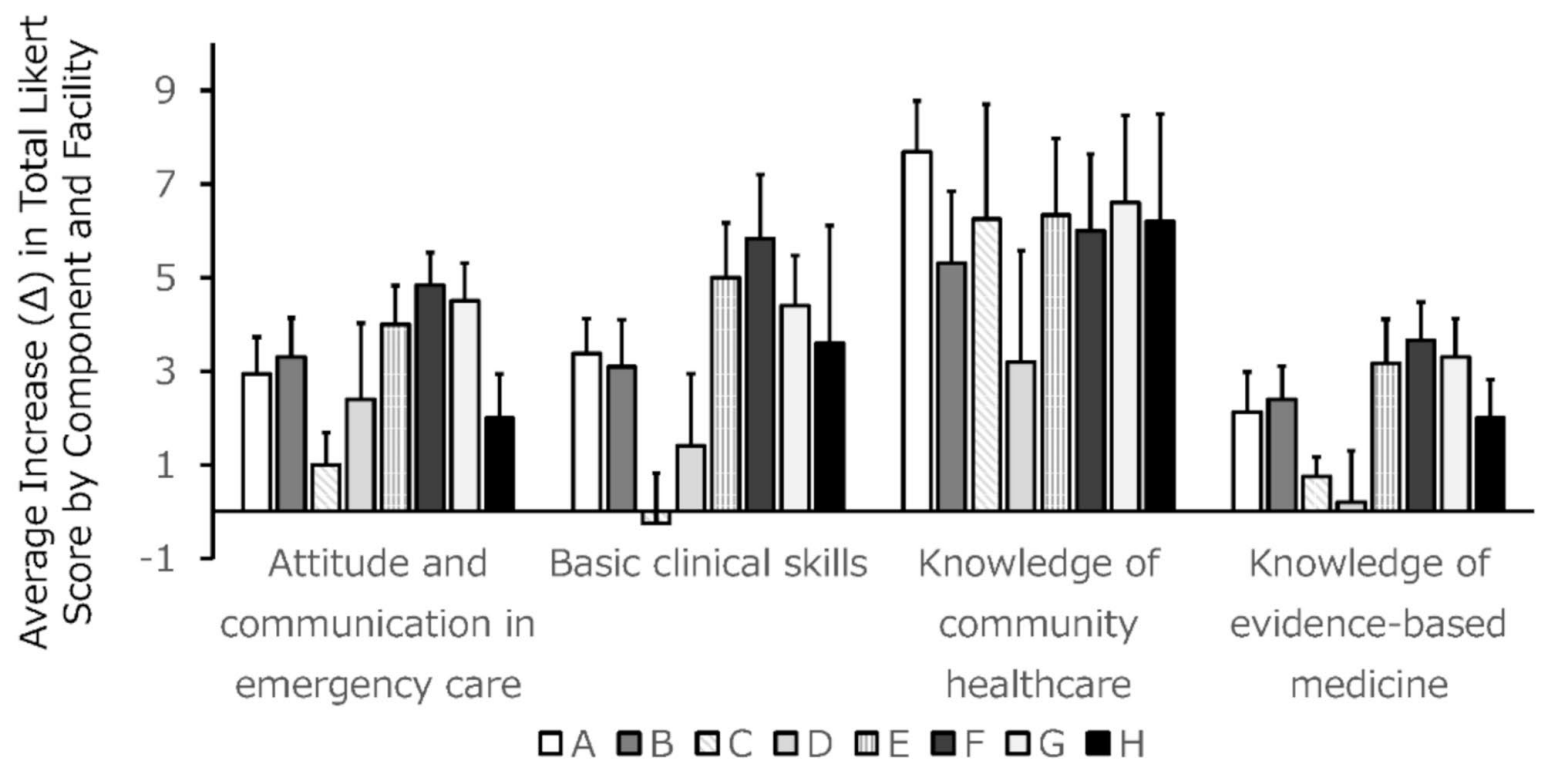
Changes in total Likert scores (total average increase; D) for the four C-CEP components, by facility (facilities A to H). Facility C does not accept emergency patients and focuses on rehabilitation and home visit consultations

There were no significant differences in the total pre-clerkship C-CEP scores across facilities (Fig. 3). However, the post-clerkship C-CEP score improvements varied among facilities (Fig. 4) Univariate (Table 3) and multivariate analysis revealed a statistically significant model (R² = 0.20, adjusted R² = 0.11, *p* = 0.0379) (Table 4). From the analysis, significant associations were observed for Facility D (estimate = -6.4, *p* = 0.0209) and Facility F (estimate = 5.9, *p* = 0.0413) (Table 4). These findings indicate that Facilities D and F exhibit notable facility-specific effects compared to the reference category. The intercept was highly significant (*p* < 0.0001), indicating a consistent baseline effect. Other facilities did not show significant associations. Other factors, such as the size of the population served, distance from Sapporo, emergency acceptance status, and number of beds, showed no significant contribution to the outcomes. Analysis of variance confirmed the model’s statistical significance (F(7, 66) = 2.29, *p* = 0.0379).

**Table 3:** Results of the univariable analysis

		*n*	Average	Standard error	*p*-value
Facility	A	19	17.4	2.0	0.04
	B	11	14.6	2.6	
	C	4	7.8	4.3	
	D	9	8.7	2.9	
	E	6	18.5	3.5	
	F	8	21.0	3.0	
	G	12	18.8	2.5	
	H	5	13.8	3.8	
Bed numbers	< 200	39	15.2	8.5	0.49
	> 200	35	16.7	9.7	
ER	present	70	16.3	9.0	0.07
	absent	4	7.8	6.9	
Distance from medical school (Sapporo) (km)	0	8	21.0	4.4	0.18
	< 100	23	16.8	8.9	
	100–200	15	12.6	10.6	
	> 200	28	15.4	8.9	
Population of city/town (thousands)	< 30	56	15.4	1.2	0.22
	30–300	10	14.2	2.8	
	< 300	8	21.0	3.2	

**Table 4:** Results of the multivariable analysis. Note: facility H served as the reference category in the regression model and is not displayed in the table

Variable	Estimate	Standard error	t-value	*p*-value
Facility [A]	2.3	2.0	1.15	0.2525
Facility [B]	-0.4	2.5	-0.18	0.8607
Facility [C]	-7.3	3.9	-1.89	0.0627
Facility [D]	-6.4	2.7	-2.37	0.0209*
Facility [E]	3.4	3.2	1.06	0.2920
Facility [F]	5.9	2.8	2.08	0.0413*
Facility [G]	3.8	2.4	1.56	0.1239
Bed	0	0	.	.
ER	0	0	.	.
Distance from Sapporo	0	0	.	.
Population of City	0	0	.	.

Note: Variables marked as “fixed to zero” were excluded from the estimation due to model constraints. Significant results are marked with an asterisk (*)

### Text mining of reflective portfolios

**Fig. 5:**
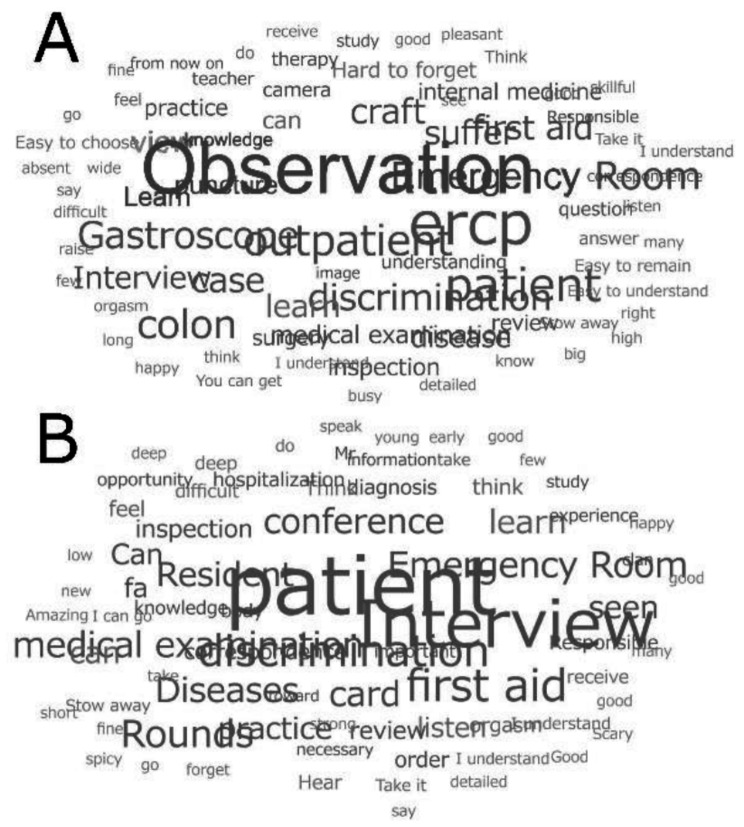
Word clouds generated from medical students’ reflective portfolios at Facility D (**A**) and Facility F (**B**), based on high-frequency scores after excluding low-informational common terms. The visualizations highlight institution-specific lexical patterns

Text mining of portfolio descriptions identified frequent nouns such as “patient” and verbs such as “go” and “think” (Tables 5 and 6). Facility F emphasized activities such as “interviews” and “examinations” in “emergency” situations and participation in “conferences”, while Facility D, which showed minimal improvement, frequently mentioned “observation”, “examination”, and “think”. The differences in reflective language suggested that Facility F provided more active learning experiences compared to Facility D. Portfolio examples from Facilities D and F are presented in Table 7. Figure 5A and B show word clouds derived from portfolio texts submitted by trainees at Facilities D and F, respectively. In Facility D, prominent terms included “observation” and “gastroscopy” suggesting a focus on clinical procedural engagement. In contrast, Facility F showed frequent use of terms such as “Patient”, “interview” and “conference” reflecting a more introspective and inquiry-based learning experience. These qualitative differences in lexical patterns complement the quantitative trends in C-CEP score changes.

**Table 5:** The five most frequently occurring words (nouns and verbs) in the portfolios of students who trained at facilities D and F

Facility	Top 5 Nouns (calculated with non-appearances as 0)	Top 5 Verbs (calculated with non-appearances as 0)
D (*n* = 8)	observation	can do
	patient	think
	outpatient	understand
	teacher	go
	surgery	perform
F (*n* = 7)	patient	can do
	interview	think
	emergency	ask
	response	go
	consultation	receive

**Table 6:** The top ten co-occurring words in the portfolios of students who completed training at facilities D and F

Top ten co-occurring words	
D	observation observation
	endoscopy observation
	colon observation
	gastroscopy observation
	camera colon
	outpatient observation
	camera gastroscopy
	colon gastroscopy
	ERCP observation
	emergency observation
F	can do patient
	interview patient
	patient ask
	think patient
	can do interview
	response emergency
	patient patient
	interview examination
	conference attend
	interview ask

**Table 7:** Portfolio examples from facilities D and F

Category number	Category	Text from Facility D	Text from Facility F
1	Things I newly realized or accomplished today	“In the new patient outpatient clinic, I observed how the doctors used blood test data to consider potential diagnoses, and I got a glimpse of the breadth and depth of their differential diagnoses. However, I also realized that even experienced doctors sometimes seem uncertain about what is acceptable or not.”	“During the interview, I was able to ask almost all the necessary questions. I realized that some people may change their story or remember things later, so it’s important not to delay and to address issues promptly.” “It’s nice to feel your own growth, isn’t it?”
2	Things that didn’t go well or failures today (what should be improved)	“I didn’t get a chance to perform any examinations.” “I should have read more about best support care beforehand.”	“I was able to examine the chest and abdomen, but I didn’t do much of an examination of the head and neck.” “There were some casual chats and missed questions, so I wish I had asked more essential questions properly.”
3	Current feelings or emotions	“It was good to learn about the atmosphere of the new patient clinic, but that was it.” “Just observing during the clinic feels like a waste.”	“I can sense the liveliness in patients, but I find it difficult to expand the conversation on the spot.” “Compared to the beginning, I feel that I can now talk more calmly.”
4	What I want to learn in the future, hopes (specific actions)	“So I thought I should take initiative by actively looking and asking questions to the doctors.”	“I want to study emergency care and outpatient care around the chest area.” “I feel like my communication skills are improving.”

## Discussion

In this study, we defined “primary care” as comprehensive, first-contact, and continuous medical care that addresses a wide range of health issues across all age groups, irrespective of geographic location. In contrast, “rural healthcare” refers to medical practice in geographically underserved areas, which often present distinct logistical and educational challenges. Our study primarily examined community-based clinical education conducted at non-tertiary hospitals, located in either rural or peri-urban settings.

This study confirmed that the Community-based assessment for Clinical and Emergency Practice (C-CEP) tool effectively detected improvements in medical students’ confidence, particularly in emergency care, following a two-week general practice clerkship. Moreover, the C-CEP highlighted differences between training sites, based on both score improvements and reflective portfolio content, demonstrating its utility as a formative tool for program evaluation and improvement.

Text mining of reflective portfolios revealed that active participation—such as patient interviews, examinations, and interprofessional conference attendance—was strongly associated with confidence gains. In contrast, passive observation contributed less or even negatively. These findings support earlier work suggesting that experiential learning fosters stronger engagement and skill development [8, 11, 14, 16].

Importantly, learner confidence plays a key role in shaping clinical behavior and future career paths [14, 18–20]. Recent studies in Australia and Japan have shown that students with higher self-efficacy for rural healthcare are more likely to pursue careers in underserved areas [12, 13]. This suggests that building confidence is not only an educational goal but also a practical strategy for workforce distribution. Similar patterns have been observed internationally; higher career-decision self-efficacy predicts active career exploration [19–21] and higher rural-practice self-efficacy predicts actual and intended practice in small communities [13].

### Novel contributions

Unlike prior research relying primarily on surveys or qualitative assessments, our mixed-methods approach integrated quantitative C-CEP scores with portfolio text analysis. This allowed for a more nuanced understanding of how and why confidence changed across training sites. Notably, students at Facility F showed marked confidence gains, while those at Facility D experienced declines—likely reflecting the degree of active engagement permitted.

While the majority of students reported increased confidence, some experienced declines. This may reflect the transition to “conscious incompetence,” where learners become aware of their limitations through exposure [22, 23]. Such decreases should not be interpreted as failure, but as critical moments for professional growth and self-reflection.

In this context, self-assessment tools like the C-CEP can serve a dual function: monitoring educational effectiveness and identifying students who may benefit from targeted support. Prior research suggests that such tools promote self-directed learning and reflective practice, and better prepare students for clinical realities [24].

Our findings also underscore the broader educational and policy implications of measuring learner confidence. Confidence—as captured by the C-CEP—is closely linked to clinical competence, motivation, and career intentions, particularly in underserved areas [12, 13, 15]. Traditional evaluation methods, such as satisfaction surveys or task completion lists, often fall short in capturing subjective learning outcomes and may be prone to bias. In contrast, confidence-based self-assessment offers an honest and practical perspective with minimal burden on supervisors.

The variation in confidence scores across facilities reinforces the need to examine instructional design. Facility F, which fostered participatory and hands-on learning, contributed positively, while Facility D, characterized by observational learning, was associated with diminished confidence. This highlights the importance of providing authentic clinical engagement opportunities during clerkships.

Moreover, a decline in confidence during regional placements could paradoxically dissuade students from pursuing careers in community medicine [22, 23]. Thus, programs should incorporate structured support systems—such as debriefings, mentoring, and feedback—to ensure these experiences reinforce rather than hinder interest in community-oriented careers.

Future studies should clearly differentiate between the effects of rural placements and those of broader community-based training environments. Understanding these nuances will better inform educational strategies aiming to address physician maldistribution.

### Limitations

This study has several limitations. Comparisons between training sites were constrained by variations in student numbers, gender distribution, consent rates, and portfolio availability. Additionally, it was conducted at a single medical university, limiting the generalizability of findings. Multi-institutional studies, including learners at various training stages (e.g., residents, fellows), are needed to validate the broader applicability of the C-CEP.

While confidence is linked to motivation and willingness to work in underserved areas [12, 13], it remains unclear whether confidence gains translate into long-term behavioral change. Further longitudinal research is warranted.

To broaden the evaluative scope beyond acute care and emergency medicine, future studies should consider additional assessment tools to capture competencies in chronic and comprehensive care settings.

## Conclusions

Participatory activities increase medical student confidence with respect to primary care and emergency responses. The C-CEP scale effectively measured confidence improvements among fifth-year medical students following their clinical clerkships. The C-CEP scale’s ability to detect inter-facility differences highlights its potential to inform and refine clinical education programs, ensuring more targeted and effective training. The ability to capture differences between training facilities enables insights into effective training elements and supports the design and enhancement of clinical education programs. Thus, the use of the C-CEP supports the educational need to enhance learner confidence in community-based medical education.

Participatory activities play a critical role in fostering motivation for healthcare delivery in underserved and resource-limited regions. While this study demonstrated the potential value of the C-CEP in measuring the confidence-building effects of various educational interventions, further research is needed to expand its application to other institutions, explore its utility across diverse levels of medical training, and assess its long-term impact on learners’ career choices and motivation to work in remote areas.

## Electronic supplementary material

Below is the link to the electronic supplementary material.

Supplementary Material 1

## Data Availability

Data is provided within the manuscript, figures, tables or a supplementary table.

## Abbreviations

C-CEP: Community-based assessment for Clinical and Emergency Practice
ER: Emergency room
ERCP: Endoscopic retrograde cholangiopancreatography
SD: Standard deviation
SEA: Significant event analysis
