# Supplementary material for: Participation-based clinical clerkships contribute to increased medical student confidence in community emergency care: a cohort study
Source: BMC Med Educ. 2025 May 20;25:734. doi: 10.1186/s12909-025-07317-1 (PMC12093790; doi:10.1186/s12909-025-07317-1)
Supplement: Supplementary file 1 — Supplementary Material 1 [file 12909_2025_7317_MOESM1_ESM.docx]

**Supplementary Table 1**. The 15-item C-CEP self-assessment questionnaire form.

| Item number | Item |
| --- | --- |
| 1 | Understands appropriate communication between doctors and patients in the emergency setting. |
| 2 | Understands appropriate communication with medical professionals such as doctors and nurses in the emergency setting. |
| 3 | Understands appropriate communication with medical professionals from other institutions in the emergency setting. |
| 4 | Can consider diseases using appropriate physical examination techniques. |
| 5 | Can explain the necessity of tests performed in the emergency setting (blood tests, arterial blood gas analysis, X-ray, electrocardiogram, ultrasound, etc.). |
| 6 | Can explain the methods and indications for basic procedures such as blood sampling, catheterization, and puncture. |
| 7 | Understands the significance of vital signs and initial treatment and can judge whether a patient requires emergency intervention. |
| 8 | Understands which professions are involved in team medicine in the emergency setting. |
| 9 | Understands the role of medical insurance in the emergency setting. |
| 10 | Understands the role of a general practitioner in the emergency setting. |
| 11 | Can cite differences in how general practitioners work depending on the region. |
| 12 | Can list the diverse job components of a general practitioner within a single facility. |
| 13 | Understands the characteristics of health problems in the region served by each training facility. |
| 14 | Can solve clinical problems using literature, textbooks, and the internet. |
| 15 | Can explain appropriate management using evidence-based medicine for common infectious diseases such as pneumonia and lifestyle-related diseases such as hypertension and diabetes. |
